# A Systematic Review of Advances in Plant-Based Phospholipid Liposomes in Breast Cancer Therapy: Characterization, Innovations, Clinical Applications, and Future Directions

**DOI:** 10.3390/ph18091288

**Published:** 2025-08-28

**Authors:** Marwa Alawi, Najihah Mohd Hashim, Noraini Ahmad, Syed Mahmood, Yi Ge

**Affiliations:** 1 Department of Pharmaceutical Chemistry, Faculty of Pharmacy, Universiti Malaya, Kuala Lumpur 50603, Malaysianajihahmh@um.edu.my (N.M.H.); 2 Centre of the Natural Products Research and Drug Discovery, Universiti Malaya, Kuala Lumpur 50603, Malaysia; 3 Computer Aided-Drug Discovery and Informatics, Faculty of Pharmacy, Universiti Malaya, Kuala Lumpur 50603, Malaysia; 4 Neuroscience Research Group (NeuRG), Faculty of Pharmacy, Universiti Malaya, Kuala Lumpur 50603, Malaysia; 5 Department of Chemistry, Faculty of Science, Universiti Malaya, Kuala Lumpur 50603, Malaysia; 6 Department of Pharmaceutical Technology, Faculty of Pharmacy, Universiti Malaya, Kuala Lumpur 50603, Malaysia; 7 Faculty of Pharmaceutical Sciences, Chulalongkorn University, Pathum Wan, Bangkok 10330, Thailand; 8 Universiti Malaya-Research Centre for Biopharmaceuticals and Advanced Therapeutics (UBAT), Department of Pharmacology, Faculty of Medicine, Universiti Malaya, Kuala Lumpur 50603, Malaysia; 9 Centre of Advanced Materials (CAM), Faculty of Engineering, Universiti Malaya, Kuala Lumpur 50603, Malaysia; 10 School of Pharmacy, Queen’s University Belfast, Belfast BT9 7BL, UK

**Keywords:** breast cancer, drug delivery, plant-based phospholipid, liposomes

## Abstract

**Introduction:** Plant-based phospholipid (PP) liposomes are sustainable, biocompatible, and biodegradable carriers with advantages over synthetic and animal-derived lipids, including lower immunogenic risk and abundant availability from sources such as soy, sunflower, and canola. This systematic review examines their characteristics, innovations, and applications in breast cancer (BCA) therapy. **Methods**: A total of 43 studies published between 2010 and June 2025 were identified from MEDLINE, Scopus, and Web of Science, focusing on PP composition, drug delivery mechanisms, and therapeutic efficacy in in vitro, in vivo, and preclinical BCA models. **Results**: Advances include nanotechnology and ligand-targeted systems that improve stability, control drug release, and enhance tumor-specific uptake. PP liposomes co-loaded with chemotherapeutics showed synergistic anticancer effects, increased tumor accumulation, and reduced systemic toxicity. Personalized targeting strategies further improved therapeutic precision and minimized off-target effects. **Conclusions**: PP liposomes offer an innovative and environmentally sustainable approach for BCA treatment with demonstrated preclinical benefits in efficacy and safety. Translation to clinical practice requires standardized characterization, scalable production, and well-designed trials to confirm safety, dosing, and long-term effectiveness.

## 1. Introduction

Phospholipids are important biomolecules that act as the cellular membranes’ building blocks. They have an amphiphilic nature as they are made of a hydrophilic, water-attracting phosphate head and two hydrophobic, water-repelling fatty acid tails [1]. This allows them to form the basis of the cell membranes as they are able to self-assemble into bilayers and play an important role in lipid structures [2]. In biological systems, phospholipids play significant roles in maintaining cellular integrity, cell signaling, membrane fluidity, and transporting molecules across membranes [3].

Phospholipids are synthesized naturally in both animals and plants; however, plant-based phospholipids (PPs) are evident as a sustainable and eco-friendly alternative to animal-derived phospholipids [4]. The most widely studied PPs are those extracted from sources like soybeans and palm oil. Unlike animal-derived phospholipids, PPs offer distinct advantages such as lower immunogenic risk, ethical sourcing, and cost-effective scalability. Their abundance in agricultural by-products like soybean or sunflower oil supports sustainable production. Moreover, PPs often have higher unsaturated fatty acid content, which may enhance membrane fluidity and improve encapsulation efficiency in drug delivery applications [5]. The current trend is an increasing focus on natural, plant-based sources due to the high demand for renewable and biocompatible materials, especially in the pharmaceutical and cosmetic industries [6].

Phospholipids have been extensively studied for their potential in the prevention and management of chronic diseases, including cancer, cardiovascular disease, diabetes, and neurodegenerative disorders [7]. The versatility in their structure forms the foundation of novel drug delivery systems aimed to improve the therapeutic effects of treatments and bioavailability for these conditions. In chronic diseases, the necessity for controlled, prolonged release of therapeutic agents is important in symptom management and slowing disease progression. This makes phospholipids important in the formulation of advanced delivery systems [8].

Among common chronic diseases, cancer is one of the leading causes of morbidity and mortality. According to Global Cancer Statistics 2022, cancer incidence was about 20 million, and 9.7 million people died due to cancer [9]. These global statistics lead to a significant advancement in cancer treatment, particularly with the evolution of drug delivery systems for efficacy enhancement and improving the precision of chemotherapeutic agents [10]. The conventional treatment of cancer, such as chemotherapy, is highly prone to non-specific toxicity, in addition to limited drug targeting and rapid drug clearance. These conditions enhanced the side effects and further reduced the efficacy of the treatment efficacy. Phospholipid-based systems, particularly liposomes, offer a solution by improving drug encapsulation, protecting the drug from degradation, and enabling targeted delivery directly to cancerous cells [11]. Phospholipid bilayers mimic natural cellular membranes, allowing them to efficiently fuse with cancer cells and deliver the therapeutic agents directly into the tumor environment [12,13].

Globally, breast cancer (BCA) is the most prevalent among women, comprising almost 25% of all cancers in women [14]. In some countries, BCA is also one of the leading causes of cancer-related deaths, such as Fiji and Jamaica [15]. The World Health Organization reported that global BCA incidence rate surpassed lung cancer and possessed an extreme impact on both high- and low-income countries [15]. Innovations in hormonal, targeted, and immunotherapies have significantly advanced breast cancer treatment, with many BCA-specific drugs having fewer side effects compared to traditional chemotherapy and offering survival benefits [16]. Despite advancements in treatment and early detection, BCA still appears with significant health challenges, due to its recurrent and aggressive forms. As one of the solid tumors, BCA consistently reported significant challenges to systemic therapy [17]. The most discussed challenges are due to inherent barriers that limit effective drug penetration in necrotic regions of the tumor, described by the irregular supply of vascular within the tumor mass and high interstitial pressures. Tumor angiogenesis promotes the formation of structurally and functionally abnormal blood vessels, resulting in increased permeability of the vessel walls [18]. These conditions disrupt the delivery systems due to limited diffusion rates of macromolecule agents within the dense tumor microenvironment [19]. Innovative drug delivery systems have been specifically designed to influence a unique physiological feature of the tumor microenvironment, which is known as the “enhanced permeability and retention” (EPR) effect. This passive targeting approach assists in addressing some of the limitations associated with conventional drug delivery methods in solid tumors [20].

This signifies the need for targeted treatment incorporated with innovation, particularly to reduce wide-ranging toxicity and minimize side effects compared to systemic chemotherapy [21]. The current trend in BCA treatment is shifting towards natural-source therapies as an alternative treatment, with abundant scientific evidence in plant-based compounds and bioactive molecules reflecting a growing demand for safer, more holistic strategies for cancer management [22]. One of the promising approaches with convincing scientific evidence to improve BCA treatment outcomes is the liposome-based drug delivery system, which uses PPs. PPs that formulate liposomes are capable of encapsulating both hydrophobic and hydrophilic anticancer drugs, increasing the drugs’ stability in the bloodstream, and facilitating targeted delivery to cancerous sites [23]. Particularly in BCA treatment, liposomal encapsulation of chemotherapy agents such as doxorubicin has been shown to increase drug accumulation within tumor tissues while minimizing exposure to normal or healthy cells [24].

Moreover, these liposomes can evade rapid clearance by the immune system, extending their circulation time and enhancing accumulation in tumor tissues through the EPR effect. This targeted delivery strategy reduces the need for higher drug doses, thereby limiting systemic toxicity and side effects, improving treatment tolerance, and enhancing the quality of life of BCA cases [25]. Although the EPR effect is generally focused on passive targeting in BCA, its effectiveness can differ significantly based on tumor heterogeneity, vascularization, and stromal composition, which can further influence nanoparticle accumulation and distribution [26]. This review distinguishes itself by providing an overview of the diverse array of phospholipid polymers (PPs) used in liposome development for cancer treatment, highlighting their unique properties. It focuses on examining the physical and chemical characteristics of PPs used in liposome formulation, advancement in preparation technologies, their role in BCA treatment across clinical and preclinical studies, and addressing the challenges, limitations, and future direction in this field. This targeted focus on phospholipid polymers and their role in BCA therapy fills a critical gap in the current literature and positions this review as a timely and valuable contribution to the field.

## 2. Methods

### 2.1. Eligibility Criteria

The search was conducted to identify relevant studies on PPs used in liposome preparation and their applications in BCA treatment. The article search focused on chemical and physical properties of PPs, their use, limitations, challenges, and current advancements in their application. Studies were excluded if they focused on animal-derived phospholipids rather than plant-based sources, if they were review articles, editorials, or case reports without experimental data, or if they did not report outcomes specific to liposomal applications in breast cancer.

### 2.2. Search Strategy and Study Selection

Systematic searching was conducted from January 2010 to June 2025 using the following databases: MEDLINE, Scopus, and Clarivate Analytics Web of Science. Related MeSH terms and keywords were searched and reported in Table 1. To identify any studies that might have been overlooked in our electronic search, we conducted supplementary searches in relevant reviews and scanned the reference lists of the included studies. Two reviewers, Marwa Alawi and Syed Mahmood, independently screened the eligible publications by title and abstract. Duplicate articles were removed, and the same reviewers screened the remaining full-text articles. Any discrepancies were resolved through discussion. The current study was designed and reported based on the guidelines of the Preferred Reporting Items for Systematic Reviews and Meta-Analyses (PRISMA).

## 3. Results

### 3.1. Data Extraction and Quality Assessed

**Figure 1:**
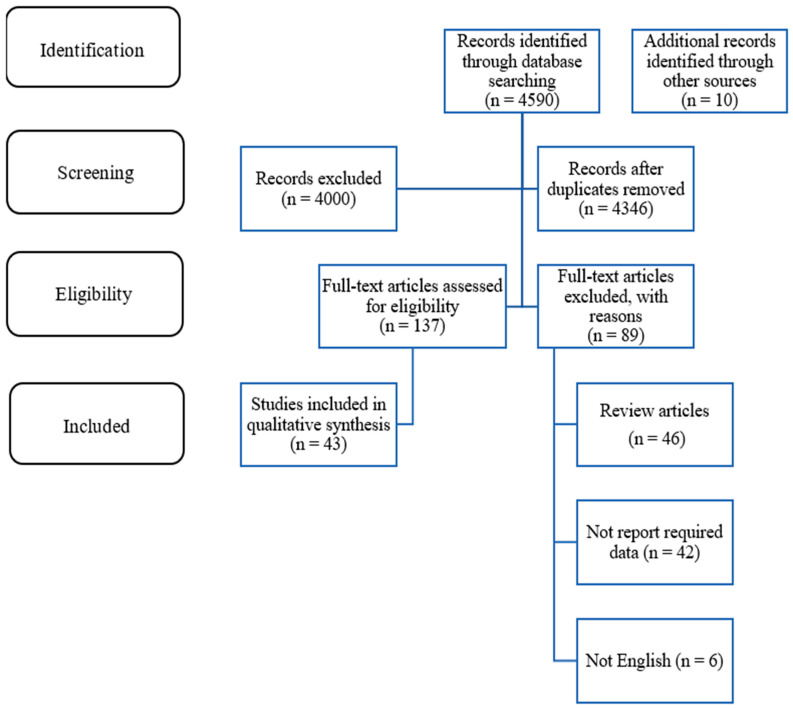
PRISMA flow diagram.

Two investigators, Marwa Alawi and Najihah Mohd Hashim, independently extracted the following data: first author name, year of publication, study design, number of subjects included if applicable, kind of plant-based, and findings. The flow of the process is summarized in Figure 1. Two researchers (Marwa Alawi and Najihah Mohd Hashim) independently assessed the quality of each included study. Disparities were resolved through consultation or by involving a third researcher (Noraini Ahmad). Quality was assessed using the Cochrane Collaboration tool 1 (ROB 1), evaluating the following seven domains: (i) random sequence generation (selection bias), (ii) allocation concealment (selection bias), (iii) blinding of participants and personnel (performance bias), (iv) blinding of outcome assessment (detection bias), (v) incomplete outcome data (attrition bias), (vi) selective reporting (reporting bias), and (vii) other biases. Each domain was rated as low risk, unclear risk, or high risk.

### 3.2. Liposome Functionalization

**Figure 2:**
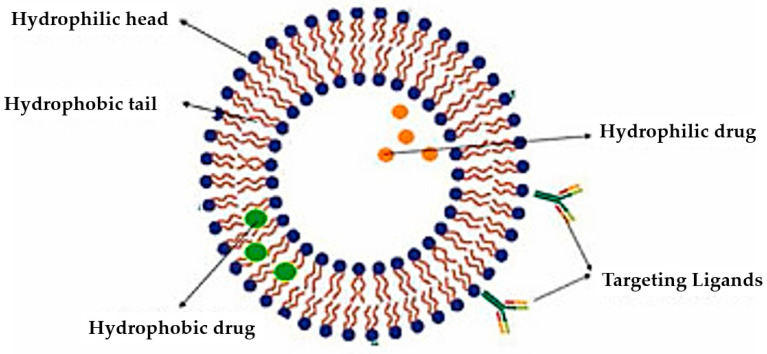
Structure of a liposome. Permission for reprint from Ajith et al. [27].

Liposomes possess a biological structure that allows them to function as drug delivery systems, with their bilayer commonly consisting of biocompatible phospholipids, sphingolipids, cholesterol, and hydrophilic polymers (Figure 2) [27]. Liposomes are adaptable drug-delivery vehicles that can also be chemically engineered to improve targeting specificity and therapeutic efficacy for cancer [28]. The functionalization is performed by conjugating targeting agents, like antibodies, peptides, or small molecules, to the liposome surface and directing them to receptors or other biomarkers present in the cancer cells [28,29]. Functionalization generally means the addition of reactive chemical groups to the liposome surface, which specifies the stable covalent binding of targeting agents through a certain type of chemical bond [30]. Different functionalization strategies include imine, amide, disulfide, thiol–maleimide, and hydrazone crosslinking, which all have their own advantages and disadvantages and are more likely to be used in a targeted drug delivery application [31]. PPs’ composition contains mainly phosphatidylcholine (PC), phosphatidylethanolamine (PE), and other minor components such as phosphatidylserine (PS) to provide a chemical handle (e.g., a primary amine or carboxylic acid) that can be used to perform covalent crosslinking (e.g., amide linkages). These natural properties can streamline surface conjugation processes without major synthetic manipulation, justifying PPs as promising, environmentally friendly candidates in functionalized nanocarrier platforms. However, it is essential to optimize reaction conditions to maintain unsaturated lipid integrity in order to maximize functionalization efficiency and stability [32].

#### 3.2.1. Imine-Crosslinked Strategy

The imine-crosslinked method employs glutaraldehyde as a crosslinker to facilitate bonding between primary amine groups on the liposome surface and aldehyde groups on the targeting agent [33]. As a bifunctional reagent, glutaraldehyde interacts with amine groups under mild conditions, forming stable imine (Schiff base) bonds [34]. This method is popular for its straightforward application and efficiency in establishing a durable connection between liposomes and biological ligands. Research indicates that imine-crosslinked liposomes have enhanced stability and controlled drug release, particularly effective in acidic environments like those in tumor tissue [35]. However, one drawback is the potential for imine bond hydrolysis under physiological conditions, which may influence drug stability and reduce targeting precision over time [36]. Therefore, this method is particularly suited for treatment requiring rapid drug release, such as in fast-growing tumors where immediate therapeutic action is essential.

#### 3.2.2. Amide-Crosslinked Strategy

Another method is the amide crosslinking between primary amine and the carboxylic acid group, forming stable covalent bonds [37]. This interaction is versatile and robust and known as amide bond or peptide bond formation, and it is commonly found in bioconjugation to attach peptides or antibodies to liposomes [38]. These bonds exhibit high resistance to hydrolysis and are stable in various physiological conditions, making them suitable for in vivo studies [39]. This is because the amine or carboxylic acid group on these biomolecules is able to form an amide bond immediately [40]. In a preclinical model of cancer, these amide-crosslink liposomes showed improvement in therapeutic outcome and targeting specificity, especially among those with long-term drug retention and required minimal leakage [41].

#### 3.2.3. Disulfide-Crosslinking Strategy

Disulfide crosslinking utilizes thiol and pyridyldithiol groups to form disulfide bonds, which are reversible and can be cleaved in the reductive environment typical of cancer cells [42]. Disulfide bonds are particularly attractive for applications in cancer therapy as they enable targeted drug release in response to the intracellular redox potential, allowing for selective drug delivery within cancer cells [43]. For instance, disulfide-crosslinked liposomes carrying doxorubicin have demonstrated enhanced cytotoxicity in breast cancer cells compared to non-functionalized liposomes, as the disulfide bonds were cleaved in the presence of high intracellular glutathione levels, triggering drug release [44]. The reversibility of disulfide bonds, however, means that these liposomes may exhibit premature drug release if exposed to reducing agents in the bloodstream, highlighting the importance of optimizing their stability for systemic administration [45].

#### 3.2.4. Thiol–Maleimide-Crosslinking Strategy

Another common and highly efficient strategy for liposome linkage is the thiol–maleimide click chemistry, as it is able to form interactions with various targeting agents such as vitamins, peptides, and antibodies [46]. This linkage strategy forms a stable thioether bond as it depends on the interaction between thiol groups on the surface of the liposome, as well as the maleimide group on the surface of the targeting agent [30]. Due to its advantage of being highly specific, thiol–maleimide chemistry can be conducted under mild conditions, and the integrity of the function can be preserved even for sensitive biomolecules during the conjugation process [47]. During antibody-conjugated liposomes, thiol–maleimide was primarily adopted to enable cancer cell precision targets that overexpress specific receptors in BCA, such as HER2 [48].

Thiol–maleimide linkage enables liposomes to co-function with various targeting agents, making them multifunctional liposomes. This directly improves therapeutic efficacy and is able to mitigate drug resistance, as these liposomes are able to target various cellular pathways simultaneously [49].

#### 3.2.5. Hydazone-Crosslinking Strategy

The interaction between aldehyde groups on the liposome and hydrazine groups on the targeting agent forms hydrazone crosslinking to form hydrazone bonds. These bonds are advantageous in physiological pH but not in acidic environments, making them effective for cancer therapy due to their high pH sensitivity [50]. They also facilitate controlled drug release in the acidic microenvironment of cancer tissue [35]. These liposomes consistently reported an enhanced anticancer activity with efficient drug accumulation within cancer cells and further reduced off-target effects [51]. For example, a selective release of drug and high tumor accumulation were reported in triple-negative BCA with liposomes functionalized with hydrazone bonds, as drug release is influenced by the acidic conditions within the tumor site, protecting the healthy tissues [52]. On the other hand, hydrazone bonds are less stable during circulation as they can be hydrolyzed at physiological pH; therefore, it is necessary to optimize their efficacy enhancement in systemic applications further [53]. This enables efficient liposomal formulations specific to patient profiles and cancer types, enabling personalized and efficacious cancer therapies [54].

There are distinct advantages and challenges for each of these functionalization strategies, which are influenced by several factors such as targeting therapeutic application, requirements, and stability considerations. Table 2 below summarizes the main features, advantages, limitations, and applications based on the specific needs of various cancer therapies. These strategies are the most promising approach in cancer treatment as they generally enable precise drug delivery and enhanced therapeutic outcomes.

Functionalizing liposomes with targeting agents through chemical modification allows for precise drug delivery and improved therapeutic outcomes, making liposomal systems one of the most promising approaches in targeted cancer treatment. As research progresses, optimization of these strategies will likely yield even. Table 2 shows a summary of the functionalization strategies in terms of chemical structure, advantages, limitations, and applications in the field.

### 3.3. Targeted Nanoliposomes for Breast Cancer Treatment

**Figure 3:**
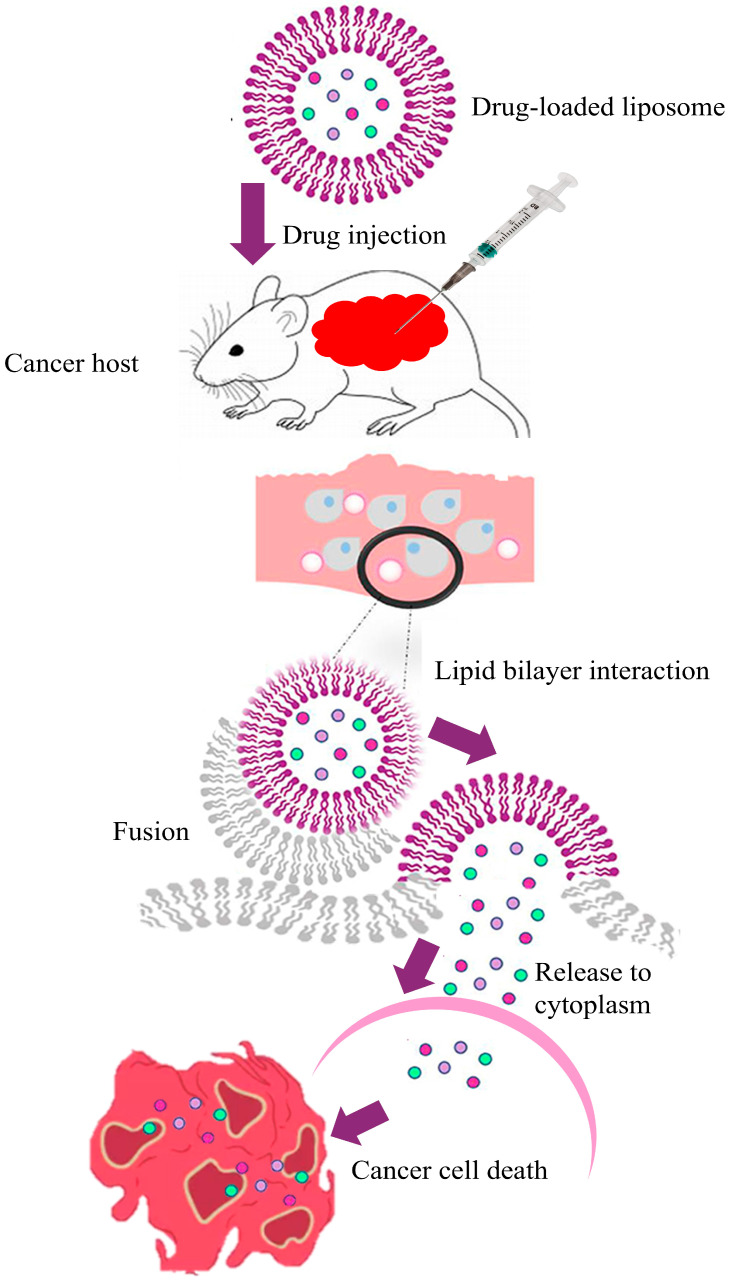
Drug administration and release pathway of nanoliposomes against breast cancer cells.

Actively targeted liposomal drug delivery systems represent a pivotal advancement in cancer therapy, especially for aggressive and recurrent forms such as breast cancer. These systems are typically engineered by decorating the liposome surface with affinity ligands (e.g., antibodies, peptides, or folate) to selectively bind receptors that are overexpressed on tumor cells (Figure 3). This receptor-mediated recognition enhances tissue retention and cellular uptake, addressing the limitations of conventional chemotherapy by improving specificity and reducing systemic toxicity [66,67]. Several studies have demonstrated the efficacy of this approach in reducing drug-related side effects and improving patient quality of life; for example, liposomal doxorubicin conjugated with targeting ligands showed a substantial reduction in cardiotoxicity while maintaining high efficacy against solid tumors [68,69]. However, recent evidence suggests that receptor engagement alone may not be sufficient to ensure effective intratumoral distribution. Beyond binding and uptake, receptor-mediated transcytosis across tumor endothelium has emerged as a distinct and crucial mechanism to achieve deeper and more uniform drug penetration within the tumor microenvironment. Li and Kataoka [70] highlighted that targeted nanomedicines designed to exploit such transcytotic pathways could overcome the heterogeneous nature of the enhanced permeability and retention (EPR) effect, ultimately improving drug accumulation and therapeutic efficacy. For plant-based phospholipid liposomes, incorporating design strategies that promote both ligand-mediated targeting and transcytotic transport could therefore represent the next frontier in maximizing clinical benefit.

Moreover, targeted liposomal drug delivery has shown potential in circumventing multidrug resistance (MDR), a formidable barrier in cancer therapy where cancer cells evolve mechanisms to expel drugs, rendering treatments ineffective [71]. MDR is often mediated by efflux pumps such as P-glycoprotein (P-gp) in cancer cell membranes, which actively remove therapeutic agents from within the cell. Targeted liposomes can bypass these efflux pumps by directly binding to specific receptors on the cell surface and delivering drugs intracellularly via receptor-mediated endocytosis, thus reducing the impact of drug resistance mechanisms [72,73,74]. In a study on breast cancer cells exhibiting MDR, targeted liposomes loaded with doxorubicin and functionalized with anti-EGFR (epidermal growth factor receptor) antibodies were able to overcome resistance and showed enhanced cellular uptake and cytotoxicity compared to non-targeted formulations [75]. By improving drug retention and accumulation within cancer cells, actively targeted liposomes hold great promise in enhancing treatment efficacy for drug-resistant tumors [76].

One additional benefit of liposomal delivery systems in cancer therapy is their potential to penetrate the blood–brain barrier (BBB), a protective layer that often restricts drug access to the brain, thus hindering treatment for brain tumors and metastases. The BBB is a tightly regulated barrier that selectively restricts the passage of substances from the bloodstream into the brain, making it challenging to deliver therapeutic agents for conditions like metastatic brain cancer [77]. Targeted liposomes designed with surface modifications, such as polysorbate-80 or transferrin receptors, can bypass the BBB by exploiting receptor-mediated transcytosis pathways [78]. For instance, transferrin receptor-targeted liposomes have demonstrated the ability to transport doxorubicin across the BBB in preclinical models, opening avenues for treating metastatic brain tumors [79]. A study by Kawak et al. [80] further highlighted the success of these systems, reporting that liposomes coated with transferrin showed a significant increase in drug accumulation within brain tumors, thereby inhibiting tumor growth more effectively than conventional chemotherapy. Studies have demonstrated that certain ligand-functionalized nanoparticles can trigger receptor-mediated transcytosis, thereby overcoming the limitations of traditional EPR-based accumulation. Incorporating such mechanisms into PP liposome design could significantly improve therapeutic delivery and warrants further exploration [74,81].

Additionally, the multifunctionality of targeted liposomal systems makes them promising candidates for a comprehensive approach to cancer treatment, encompassing not only drug delivery but also imaging and diagnostic capabilities [82]. In cases of metastatic, relapsed, and breast cancer-associated cells, these liposomes can be engineered with imaging agents, such as fluorescent dyes or radioisotopes, for diagnostic purposes [83]. This enables real-time tracking of drug distribution within the body, allowing oncologists to monitor treatment efficacy and tumor response. For example, Herceptin-conjugated liposomes carrying both doxorubicin and a fluorescent marker were utilized to track therapeutic response in HER2-positive breast cancer cells, providing insights into tumor regression and facilitating personalized treatment plans [84]. The integration of imaging capabilities into therapeutic liposomes has shown potential in preclinical studies, supporting the concept of theragnostic liposomes that combine therapeutic and diagnostic functions in a single platform, thus optimizing treatment regimens and enhancing patient outcomes [85].

Despite the numerous advantages, the development of targeted liposomal drug delivery systems is not without challenges. One of the primary obstacles is identifying viable and specific targets on cancer cells that distinguish them from healthy cells. Cancer cells often express unique or overexpressed receptors, such as folate receptors, HER2, and integrins, which can serve as docking sites for liposomes [86]. However, heterogeneity within tumors and among cancer patients can complicate the identification of universal targets, necessitating a personalized approach to treatment. Another critical consideration is the need to graft liposomes with targeting moieties, such as ligands or antibodies, without compromising the stealth properties of the liposomes [87]. Liposomes are often coated with polyethylene glycol (PEG) to evade immune detection and extend circulation time, allowing them to accumulate preferentially in tumor tissue via the enhanced permeability and retention (EPR) effect [88]. However, PEG stealth technology is not without challenges. One of the most notable issues is the accelerated blood clearance (ABC) phenomenon upon repeated dosing, driven by anti-PEG IgM/IgG production, complement activation, and rapid hepatic uptake. Other drawbacks include hypersensitivity reactions, reduced cellular uptake due to steric hindrance, instability under certain physiological conditions, and the so-called pseudo-stealth effect, in which blood concentration drops sharply within the first hour despite a prolonged β-phase. Both treatment-induced and pre-existing anti-PEG immunity can further compromise stealth performance. Recent advances aim to address these limitations through a “structural holism” approach—optimizing the entire nanocarrier surface to minimize charges, dipoles, and hydrophobic domains—alongside strategies such as mixed polymer coatings, biodegradable PEG analogs, zwitterionic or biomimetic membranes, and dynamic stealth systems that adapt surface properties in vivo. Additionally, engineering PEG-lipid derivatives, integrating high-performance nanomaterials, and leveraging pseudo-stealth deliberately to modulate RES clearance have been explored to retain PEG circulation benefits while mitigating its immunogenicity and dose-dependent pharmacokinetics [89,90,91]. The addition of targeting ligands, however, can reduce the stealth effect, making the liposomes susceptible to immune clearance. Optimizing the balance between targeting efficacy and stealth properties remains a focus of ongoing research in the field of nanomedicine [92].

Furthermore, preclinical and clinical studies have underscored both the promise and limitations of targeted nanomedicines for solid-tumor treatment [93]. While preclinical models have shown encouraging results with improved drug delivery and reduced side effects, the transition to clinical use remains challenging. Factors such as scale-up production, stability, and reproducibility must be addressed to ensure the feasibility of these systems for widespread therapeutic applications [94].

#### 3.3.1. Ligands in Targeted Liposomal Drug Delivery Systems

In the development of targeted liposomal drug delivery systems, ligands play a crucial role in directing liposomes to cancer cells while minimizing side effects to healthy tissue [11]. Common ligands include small molecules, monoclonal antibodies (mAbs), peptides, and aptamers, each with unique advantages and challenges that influence their suitability in clinical applications [95]. Initial research efforts focused on whole monoclonal antibodies due to their high specificity and affinity for target cells. However, the large size of mAbs poses a significant limitation, as it can hinder their penetration into tumor tissue and increase immunogenicity, leading to potential adverse immune responses [96]. Additionally, the cost of producing full mAbs remains a barrier to widespread use in liposomal systems [97]. Consequently, research has shifted towards smaller antibody fragments, such as fragment antigen-binding (Fab) and single-chain variable fragments (scFv), which retain the targeting ability of whole antibodies but with reduced immunogenicity and improved pharmacokinetic properties [98].

Peptides have emerged as an appealing alternative to antibodies for targeting purposes. Their low cost, ease of preparation, and relatively small size make them highly accessible for large-scale production. Moreover, peptides can be engineered to bind selectively to specific cancer cell receptors, reducing the risk of non-specific interactions [99]. However, a primary challenge with peptide ligands is their susceptibility to proteolysis, which can lead to rapid degradation in the bloodstream and limit their effective duration within the body [79]. Peptide modification, such as incorporating non-natural amino acids or using cyclization techniques, has shown promise in enhancing their stability. Despite their biocompatibility and sustainability advantages, the large-scale extraction, purification, and quality control of plant-based phospholipids remain complex and cost-sensitive, limiting widespread adoption in industrial manufacturing settings [100].

Small-molecule ligands, such as the kinase inhibitor sorafenib, are also utilized in targeted liposomal formulations due to their ease of synthesis, high stability, and cost-effectiveness [101]. However, a notable drawback of small molecules is their limited specificity, as they often interact with multiple receptor types, leading to off-target effects and reduced therapeutic efficacy [96]. Despite these limitations, small molecules can be useful for targeting applications where a broader targeting strategy is acceptable, or for use in conjunction with other, more selective ligands [102].

Aptamers, single-stranded oligonucleotides or peptides have gained attention for their high affinity and specificity towards target molecules. Aptamers are highly stable and can be readily modified for targeted delivery applications [103]. Unlike antibodies, they are non-immunogenic, which enhances their safety profile [104]. Despite these advantages, aptamers face significant challenges related to their rapid clearance from circulation and susceptibility to nuclease degradation, which limits their half-life and reduces their effectiveness in vivo [105]. Efforts to improve the pharmacokinetic profiles of aptamers, such as PEGylation or conjugation to nanoparticles, have shown promise in extending their therapeutic window, though these modifications can impact on their binding affinity and cellular uptake [106].

#### 3.3.2. Synthetic Liposomal Drug Delivery Developments in Breast Cancer

Liposomal formulations are typically characterized by their phospholipid bilayer, which can encapsulate hydrophilic drugs in the aqueous core and hydrophobic drugs within the lipid membrane [107]. The composition of liposomes can be tailored to enhance their stability, release profiles, and targeting abilities [108]. The size of liposomes typically ranges from 50 nanometer to several micrometers, affecting their biodistribution and cellular uptake [109]. Table 3 shows the composition, size, targeting strategies, and challenges of common/synthetic liposomal drug delivery systems.

#### 3.3.3. Mechanisms in Breast Cancer

Various targeting mechanisms were addressed in liposomal drug delivery to improve therapeutic drug accumulation and efficacy in BCA [138]. Folate receptors can facilitate selective drug delivery by binding to cancer cells, while CD44 is a cell-surface glycoprotein involved in cell–cell interactions [139]. Folate receptors and CD44, the surface receptors, are ideal targets for liposomal formulation as they are commonly overexpressed in BCA cells [140]. HER2, the transmembrane receptor, also plays an important role in BCA progression. Liposomes formulated with ligands or HER2-specific antibodies provide a more targeted therapeutic approach as they are able to improve drug accumulation in HER2-positive tumors [141].

Enzyme-responsive liposomes target internal cell receptors, which signifies another innovative strategy. These liposomes are formulated to react to specific enzymes that are common in tumor microenvironments and further enable controlled drug release. For example, matrix metalloproteinases (MMPs), which are commonly overexpressed in cancers, are able to cleave peptide linkers in liposomal formulations, inducing drug release at the cancer site [142]. Dual-targeting strategies were also introduced in liposomal drug delivery systems with simultaneous targeting of both surface and internal receptors, such as CD44 and MMPs. These strategies enhanced the therapeutic outcomes as it was reported to be able to improve drug accumulation and cancer cell uptake [143].

#### 3.3.4. Cell Surface, Transmembrane, Internal Cell, and Enzyme Receptors in Liposomal Drug Delivery Systems for Breast Cancer

Liposomal drug delivery systems have emerged as a promising approach for enhancing the targeted treatment of cancer [144]. By exploiting the unique characteristics of various cell surface receptors, these systems can improve drug accumulation in tumor cells, address drug resistance, and enhance therapeutic efficacy [145]. Table 4 reviews key cell surface receptors relevant to breast cancer and how liposomal formulations can be designed to target these receptors effectively. Each receptor not only plays a significant role in breast cancer progression but also has supporting evidence from previous studies that validates its potential as a target for liposomal drug delivery systems [11,144]. Previous study findings provide evidence that targeted approaches can improve therapeutic outcomes and address challenges in breast cancer treatment [146,147].

### 3.4. Plant-Based Phospholipids

Plant-based phospholipids (PPs) are a diverse lipid group that have suitable structures for applications in various fields, including drug delivery, food processing, and cosmetics [169]. Phospholipids’ unique amphiphilic properties, which are characterized by hydrophilic (water-attracting) as well as hydrophobic (water-repelling) regions, allow for self-assembly into bilayers and form liposomes. These characteristics are essential for encapsulating and delivering active ingredients to specific targets. In drug delivery, phospholipids increase the stability and bioavailability of drugs, at the same time facilitating controlled release of the drug and enhancing the effectiveness of the therapy [170]. In cancer treatment and management, PP liposomes enable targeted drug delivery as they enhance the permeability and retention (EPR) effect in the cancer cells. Evidence consistently shows that targeted strategies are the best approach to reduce off-target toxicity and side effects. These features of PP liposomes make them more patient-friendly compared to conventional or synthetic drug formulations. Moreover, PPs are more immunogenic and have minimal to nil adverse immune responses. Table 5 is an overview of common types, sources, chemical structures, and their application. Table 6 illustrates the physical properties of the PPs, while Table 7 summarizes the chemical properties of PPs.

## 4. Discussion

Previous studies on the use of PP liposomes with chemotherapeutic drugs for BCA treatment are summarized in Table 8. One of the most notable examples is Doxil, a pegylated liposomal formulation of doxorubicin, which has been successfully utilized in the treatment of breast cancer [217,218,219,220,221,222]. Studies have demonstrated that Doxil significantly reduces the cardiotoxicity associated with free doxorubicin while maintaining efficacy in tumor reduction [221]. Other formulations, such as Myocet (liposomal doxorubicin), have also shown promise in clinical trials, emphasizing the safety and effectiveness of liposomal drug delivery systems [119,120,121]. Recent investigations have focused on the incorporation of plant-based phospholipids in liposomal formulations, exploring their potential in breast cancer treatment. These formulations leverage the biocompatibility and biodegradable properties of natural phospholipids derived from sources such as soy, palm oil, sunflower, etc. [218]. The review showed that the use of PPs offers enhanced drug delivery, reduces systemic toxicity, and enhances therapeutic outcomes compared to chemotherapeutic agents. At the molecular level, PPs improve drug encapsulation, stability, and targeted intracellular delivery, increasing uptake by cancer cells and greater endosomal escape, leading to enhanced DNA intercalation and apoptosis [72,79,96]. The ability for targeted delivery was enhanced by this liposomal system [96,167]. Additionally, the targeted delivery capability of these liposomal systems facilitates increased drug accumulation at the tumor cells without harming the normal cell [75]. PPs showed a better safety profile and higher tolerance due to their natural composition and reduce the chance or event of adverse reaction [47]. Studies on soy-derived phospholipids, sunflower-derived phosphatidylcholine liposomes, curcumin, green tea, and resveratrol showed therapeutic benefits through improved drug accumulation within the tumor site, minimizing systemic toxicity, enhancing apoptosis, inhibiting proliferation, and enhancing anticancer effects. Studies using palm-oil-derived phospholipid as a liposome also consistently showed therapeutic benefits such as high bioavailability, minimizing cardiotoxicity, and enhanced chemotherapy effects. These findings suggest that PP liposomes are an effective strategy in drug delivery and are highly compatible with various chemotherapy drugs. The usage of PP liposome is able to address various conventional challenges, including drug resistance, toxicity, overdose, and off-target effects [159,160,163,166,167,223].

There is a need for future research to include clinical trials to confirm the long-term safety potential and therapeutic effect of PP liposomes across various populations. Additionally, to translate these findings into approved BCA treatment strategies, further clinical and preclinical studies are required. Aspects such as stability at different physiological conditions, molecular mechanisms, and genetic expression should be assessed to evaluate the effectiveness of these PP liposomes in combination with conventional drugs to enhance personalized BCA therapeutic strategies.

PP liposomes show strong potential for clinical application, particularly in BCA, due to their biocompatibility and natural origin. While regulatory and manufacturing challenges remain, such as ensuring consistency and meeting quality standards, ongoing advances in lipid characterization, process optimization, and surface engineering (e.g., PEGylation or ligand targeting) are helping to overcome these barriers. Another key issue is batch-to-batch variations in composition based on plant source, extraction protocols, and seasonal or geographic variation. This variability could affect the physicochemical properties of liposomes, such as the encapsulation efficiency, stability, and delivery profiles of the controlled drug release. As an example, changes in membrane fluidity brought about by changes in saturation levels of fatty acids can influence the release kinetics of an encapsulated drug. Moreover, though PPs are typically viewed as less immunogenic than those of animal origin, few direct comparative studies have evaluated immunologic responses to the two materials. In the absence of standardized immunological profiling, the inference of reduced immunogenicity is hypothetical. A rigorous assessment is required to identify whether PP liposomes truly elicit lesser immune responses in clinical trials than animal-derived preparations. Early in vivo studies highlight promising biodistribution and safety profiles, yet further human data are needed. Strengthening pharmacokinetic evidence and standardization strategies will support the clinical translation of PP liposome systems [224].

**Table 8:** Usage of plant-based phospholipids as liposomes with chemotherapeutic agents in breast cancer treatment.

Study	Study Type	Liposomal Composition	Chemotherapeutic Agent	Key Findings
Coutinho et al., 2023 [225]	Preclinical	Soy-derived phospholipids; genistein incorporated in phosphatidylcholine–cholesterol liposomes	Paclitaxel	Enhanced drug accumulation in tumor tissues; improved therapeutic outcomes compared to free paclitaxel.
Lo et al., 2024 [226]	Preclinical	Sunflower-derived phosphatidylcholine liposomes	Doxorubicin	Significant tumor regression and reduced systemic toxicity; demonstrated safety and efficacy of plant-based liposomes.
Chavda et al., 2023 [227]	Preclinical	Phosphatidylcholine (PC) with natural surfactants	Curcumin combined with chemotherapy	Enhanced anticancer effects of curcumin; improved response rates in breast cancer models.
Meng et al., 2016 [228]	Preclinical	Phosphatidylcholine–cholesterol liposomes co-encapsulating resveratrol (RSV) and paclitaxel (PTX)	RSV + PTX	Synergistic anticancer effects; enhanced efficacy versus single-drug formulations.
de Pace et al., 2013 [229]	In vitro	Soy lecithin–cholesterol liposomes coated with chitosan (CSLIPO-EGCG)	Green tea-Epigallocatechin-3-gallate (EGCG)	Enhanced apoptosis and proliferation inhibition in MCF7 cells; effective at ≤10 μM; chitosan coating improved stability and reduced immunogenicity.
Yousfan et al., 2024 [230]	Preclinical	Lipid droplet from date palm seed (DPLDs)	Paclitaxel	Enhancement of solubility, reduction in toxicity, and improved brain accumulation of paclitaxel via intranasal delivery.
Li et al., (2020) [231]	Preclinical	Corosolic acid liposomes (CALP) from Lagerstroemia speciosa, cholesterol-free	Doxorubicin	Higher cellular uptake, tumor penetration, and accumulation; significant tumor growth inhibition and extended survival in 4T1 murine model; high anti-inflammatory activity via STAT3 inhibition.
Tang et al., (2014) [222]	Preclinical	Platycodon (PD) derived from the Platycodon grandiflorum plant (balloon flower)	Doxorubicin	DOX/PD showed enhanced anti-proliferative effects on n human breast cancer cell lines (MCF-7 and MDA-MB-231). Higher apoptosis-related protein expression (e.g., cleaved PARP). Reduction in mitochondrial membrane potential. Higher intracellular accumulation of DOX in MDA-MB-231 (a triple-negative breast cancer line).
Sabeti et al., (2014) [219]	Preclinical	Phosphatidylcholine liposomes containing 5% or 10% palm oil	Doxorubicin	Higher encapsulation efficiency; sustained release; lower IC50 in MCF-7 and MDA-MB-231 cells.
Bedretdinov & Kostryukova, 2023 [218]	Preclinical	Palm oil phospholipids	Doxorubicin	Higher cytotoxicity against MDA-MB-231 cells, stable particle size, and zeta potential contributed to efficacy.
He et al., 2023 [232]	Preclinical	PLGA nanoparticles loaded with palmitic acid (from palm oil)	Doxorubicin	Reduced cell viability and migration in vitro; decreased tumor growth and metastasis in vivo; immunomodulatory effect via macrophage polarization.
Franco et al., 2019 [233]	Preclinical	Palm oil phospholipid liposomes	Paclitaxel + Doxorubicin	Higher tumor inhibition ratios in 4T1 breast cancer cell line. Improved cardiac toxicity profile.
Mahmoudi et al., 2021 [234]	Preclinical	Curcumin from plant Curcuma longa (turmeric)	Cisplatin	High entrapment efficiency Significant higher cytotoxicity (82.5%) and lowered breast cancer cell viability. Tenfold increase in apoptosis.
Sunoqrot et al., 2023 [217]	Preclinical	Polyquercetin (pQCT), a plant-derived polymer based on quercetin	Doxorubicin	Exhibited spherical morphology, high monodispersity, excellent drug loading capacity, and sustained drug release. Enhance induce apoptosis in breast cancer cell.
Chavoshi et al., 2023 [220]	Preclinical	Soybean lecithin–cholesterol liposomes loaded with crocin (from Crocus sativus)	Doxorubicin	Dose-dependent enhanced cytotoxicity in triple-negative breast cancer (TNBC) cells. Induced cell cycle arrest at Sub-G1 and G2/M phases. Downregulated anti-apoptotic genes (survivin, cyclin-B1, Bcl-xl). Upregulated pro-apoptotic genes (Bax, Bid). Better apoptotic and antiproliferative properties.
Pandey et al., 2025 [235]	Preclinical	Folic acid (FA)	5-Fluorouracil (5-FU)	Enhanced uptake, cytotoxicity, and migration inhibition in MCF-7 breast cancer cells. Enhanced apoptosis. Superior tumor growth inhibition. Lower systemic toxicity, and improved overall safety profile compared to conventional 5-FU formulations.
Eloy et al., (2017) [236]	Preclinical	Anti-HER2 immunoliposomes	Paclitaxel + Rapamycin	Higher cytotoxicity in HER2-positive SKBR3 cells; reduced tumor volume to 25.27% of control.
Pogorzelska et al., 2023 [221]	Preclinical	Sulforaphane, a naturally occurring isothiocyanate found in cruciferous vegetables like broccoli	Doxorubicin	Sulforaphane enhanced nuclear accumulation of DOX. Twofold inhibition of tumor growth observed in vivo. Potential to reduce DOX dose fourfold while maintaining efficacy due to synergistic interaction. Sulforaphane inhibited mitosis in cancer cells. Protected normal cells by displaying antagonistic cytotoxicity. Reduced cardiotoxicity, nephrotoxicity, and hepatotoxicity in vivo.
Attia et al., 2025 [223]	Preclinical	Alpha-lipoic acid (ALA) and ascorbic acid (AA)	Doxorubicin	Biphasic drug release. Effective cytotoxic activity against breast cancer cell lines. Reduced nephrotoxicity.
Vakilinezhad et al., 2019 [237]	Preclinical	PLGA nanoparticles co-loaded with curcumin (Curcuma longa) and methotrexate	Methotrexate	Enhanced cytotoxicity against breast cancer cells. Improved targeted delivery, controlled release, and reduced systemic toxicity.

## 5. Conclusions

The current review reveals an important innovation gap, that is, the absence of uniform protocols and strong clinical evidence regarding PP liposomes as BCA therapy. Although the reported preclinical results demonstrate improved drug delivery, stability, and minimized side effects, the safety, efficacy, dosage, and delivery protocol but to be translated into clinical application; there is still a need to confirm these findings through randomized controlled trials (RCTs) to further translate into sustainable, patient-friendly, and targeted treatment approaches with global health and sustainability objectives. Key barriers include the lack of standardized extraction methods, batch-to-batch consistency, and robust immunogenicity data. Future efforts should prioritize well-designed RCTs, scalable manufacturing, and integration with combination and personalized therapies to establish PP liposomes as clinically viable and sustainable options for breast cancer management.

## Figures and Tables

**Table 1:** Search strategy according to sections.

Keywords/Search Terms
(“Breast Cancer” AND “Treatment” OR “Management” OR “Therapy”) AND (“Surgery” OR “Chemotherapy” OR “Radiation” OR “Targeted Therapy” OR “Hormonal Therapy” OR “Drug Delivery”)AND (“Plant-Based Phospholipids” OR “Plant-Derived Phospholipids” OR “Palm Oil” OR “Soy Lecithin” OR “Sunflower Lecithin) AND (“Liposomes” OR “Encapsulation”) AND (“Case Study” OR “Case Report” OR “Clinical Trial” OR “Patient Outcome”)

**Table 2:** Characteristics of liposome functionalization.

Functionalization Strategy	Chemistry Feature	Advantages	Mechanisms of Action	Limitations	Example Applications	References
Imine-crosslinked	Glutaraldehyde-mediated linkage between aldehyde and primary amine	Simple, efficient, reversible bonding	Short-term release in mildly acidic tumor microenvironment (pH 5–6); enables nanoparticle or hydrogel surface functionalization via reversible imine bond formation	Hydrolysis-prone at physiological pH; potential cytotoxicity of glutaraldehyde	Tumor-targeted short-term drug release	[55]
Amide-crosslinked	Primary amine and carboxylic acid (often EDC/NHS-mediated)	Stable, robust in vivo covalent linkage	Durable bond for ligand (e.g., antibody/peptide) attachment; supports active targeting of tumor antigens	Requires available carboxylic group; coupling chemistry may require activation	Antibody–drug conjugates, peptide–nanoparticle conjugates	[56,57,58]
Disulfide-crosslinked	Thiol and pyridyldithiol exchange reaction	Redox-responsive release	Facilitates drug release inside tumor cells with high glutathione levels; offers intracellular specificity	Susceptible to premature cleavage in bloodstream due to reductants	Intracellular cancer drug delivery	[59,60,61]
Thiol–maleimide	Michael-type addition between thiol and maleimide	Specific, mild conditions	Enables site-specific conjugation of targeting ligands; supports efficient dual-ligand targeting	Potential thiol exchange or instability in vivo	Antibody–nanoparticle conjugation, dual-ligand targeting systems	[62,63]
Hydrazone-crosslinked	Aldehyde and hydrazine	pH-sensitive release in acidic tumor microenvironment	Hydrazone linker cleaves under mildly acidic conditions (pH 5–6), enabling controlled drug release	Hydrolysis at neutral pH can occur	Acidic tumor microenvironment-targeted drug release	[64,65]

**Table 3:** Composition, size, targeting strategies, and challenges of common/synthetic liposomal drug delivery systems.

Liposomal System	Name of Drugs	Composition	Size (nm)	Targeting Strategy	Advantages	Limitations	Approval Status	References
Doxil^®^/Caelyx^®^	Doxorubicin	PEGylated liposomes	~100	Passive (EPR effect via PEGylation)	Long circulation time, reduced cardiotoxicity	Hand–foot syndrome, stomatitis	Approved	[110]
Onivyde^®^	Irinotecan	Lipid bilayers encapsulating irinotecan	~110	Passive (EPR)	Improved stability, prolonged circulation	No active targeting	Approved	[111,112,113]
HER2-immunoliposome (investigational)	Doxorubicin	Cholesterol, DSPC, DSPE-PEG2000 with anti-HER2 antibody	70–150	Active (HER2)	Enhanced specificity for HER2+ tumors	Investigational, potential immunogenicity	Investigational	[114,115,116]
Lipusu^®^	Paclitaxel	Phosphatidylcholine, cholesterol	100–200	Passive (EPR)	Improved pharmacokinetics	High manufacturing costs	Approved (China)	[117,118]
Myocet^®^	Doxorubicin	Non-PEGylated liposomes (PC, cholesterol)	150–200	Passive (EPR)	Reduced cardiotoxicity vs. free DOX	Shorter circulation than PEGylated forms	Approved (EU, Canada)	[119,120,121]
Marqibo^®^	Vinorelbine	Sphingomyelin, Cholesterol	~100	Passive (EPR)	Improved stability, enhanced delivery to tumors	No active targeting, potential off-site accumulation	Approved (US)	[122,123,124]
Lipoplatin^®^	Cisplatin	Dipalmitoyl phosphatidyl glycerol (DPPG), soy phosphatidylcholine (SPC-3), cholesterol, polyethylene glycol (PEG)	110–120	Passive (EPR)	Reduced toxicity, prolonged circulation	Decreased recognition by the immune system.	Investigational/Limited markets	[125,126,127]
LEM-ETU (investigational)	Mitoxantrone	Egg phosphatidylcholine (EPC), cholesterol, polyethylene glycol (PEG) coating	100–150	Likely passive (no verified RNA targeting)	Enhanced tumor accumulation	Passive targeting and accumulation in off-targeted sites, hence leads to side effects in non-tumor tissues.	Investigational	[128,129]
Trastuzumab-immunoliposome	Doxorubicin	Phospholipids, cholesterol	100–150	Active (HER2)	High efficacy in HER2+ tumors	Not effective in HER2– tumors	Investigational	[27,130]
DMPC/DSPC Cholesterol liposomes	Doxorubicin	Dimyristoylphosphatidylcholine (DMPC), distearoylphosphatidylcholine (DSPC), cholesterol	100–150	Passive (EPR)	Enhanced stability, reduced cardiotoxicity	Non-specific accumulation	Research	[131,132,133]
Hyaluronic acid-liposomes	Doxorubicin and paclitaxel	Phospholipids, cholesterol	100–150	Active (CD44)	Dual drug delivery, CD44 specificity	Limited to CD44+ tumors	Investigational	[134,135]
Hyaluronic acid-liposomes	Shikonin	Phospholipids, cholesterol and hyaluronic acid (HA)	100–150	Active (CD44)	Enhanced stability, CD44 specificity	Limited to CD44+ tumors	Investigational	[136,137]

**Table 4:** Targeted cell surface receptors in liposomal drug delivery systems for breast cancer.

Cell Surface Receptor	Role in Cancer	Targeting Strategy	Potential Benefits	Relevance to Breast Cancer	Supporting Evidence
**C-X-C Chemokine Receptor Type 4 (CXCR4)** 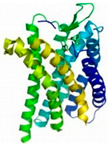	Overexpressed in breast tumors; regulates migration, invasion, and metastatic spread via CXCL12 axis.	CXCR4 antagonists (e.g., AMD3100) in liposomal or nanoparticle formulations.	Reduces metastatic dissemination and enhances therapeutic efficacy of chemotherapeutics.	Highly expressed in triple-negative breast cancer (TNBC) and linked to poor prognosis.	In TNBC xenografts, CXCR4 blockade reduced lung metastases and synergized with chemotherapy [148].
**Cell Surface Nucleosomes** 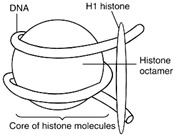	Involved in immune evasion by cancer cells.	Targeted liposomes to enhance immune response against tumors.	Promotes immune-mediated destruction of cancer cells.	Present on breast cancer cells, contributing to immune escape mechanisms.	Increased nucleosome levels correlate with poor prognosis in breast cancer patients [149].
**Eph Receptors** 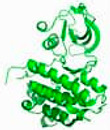	Facilitates cell signaling and tumor progression.	Liposomes designed to disrupt Eph receptor signaling.	Reduces tumor growth; interferes with proliferative signaling.	EphA2 is particularly relevant; associated with aggressive breast cancer phenotypes.	EphA2-targeted therapies have shown reduced tumor growth in xenograft models [150].
**Folate Receptor** 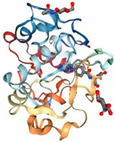	Overexpressed in many cancers, including breast cancer.	Folate-targeted liposomes for improved drug delivery.	Enhances therapeutic agent concentration in tumors; minimizes off-target effects.	High expression in breast cancer cells; improves targeting for chemotherapeutics.	Folate-targeted liposomal formulations enhanced delivery and efficacy of doxorubicin in breast cancer models [151].
**Intercellular Adhesion Molecule-1 (ICAM-1)** 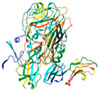	Cell adhesion molecule involved in tumor–endothelial interactions and immune cell infiltration.	Antibody–drug conjugates, imaging probes, and RNA-based silencing.	May limit metastatic colonization and facilitate immune targeting.	ICAM-1 is expressed in several solid tumors; limited direct targeting data in breast cancer.	Breast cancer imaging studies demonstrate ICAM-1 overexpression; functional blockade studies sparse [152].
**Lipoprotein Receptor-related Protein-1 (LRP-1)** 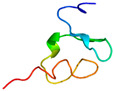	Endocytic receptor implicated in tumor cell migration, invasion, and angiogenesis.	siRNA-mediated knockdown, receptor-blocking peptides, and ligand-targeted nanocarriers.	Delays tumor growth and reduces angiogenic signaling.	Preferentially expressed in TNBC, correlating with invasive phenotype.	LRP-1 silencing in MDA-MB-231 xenografts caused ~60% tumor growth delay [153].
**Nucleolin** 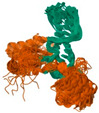	Multifunctional protein involved in ribosome biogenesis and regulation of mRNA stability; overexpressed on cancer cell surfaces.	AS1411 aptamer-based nanocarriers and targeted drug delivery systems.	Enables selective drug delivery and tumor imaging.	Highly expressed in breast cancer cells; absent or low in normal tissues.	Nucleolin-targeted nanoparticles demonstrated selective cytotoxicity in breast cancer models [154]
**P-glycoprotein (P-gp)** 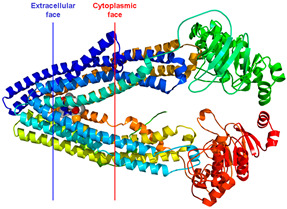	ATP-dependent efflux transporter mediating multidrug resistance.	Co-delivery of P-gp inhibitors with chemotherapeutics using nanocarriers.	Restores chemosensitivity and increases intracellular drug accumulation.	Upregulated in resistant breast cancer cell lines.	P-gp inhibition via nanoparticle delivery increased doxorubicin retention and efficacy in resistant breast cancer cells [155].
**Somatostatin Receptor** 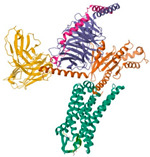	G-protein coupled receptor involved in hormone regulation and tumor growth suppression.	Radiolabeled peptides (e.g., 68Ga-DOTATATE) and SSTR2-inducing epigenetic modulators.	Improves imaging sensitivity and enables targeted radionuclide therapy.	Inducible in TNBC cells; baseline expression varies.	HDAC inhibitor pretreatment enhanced SSTR2 expression and imaging in TNBC models [156].
**Sigma Receptor** 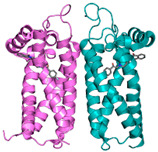	Chaperone protein regulating calcium signaling, ER stress, and cell survival.	Small-molecule antagonists and targeted nanoparticles.	Induces apoptosis and reduces tumor proliferation.	Overexpressed in breast cancer tissue and cell lines.	Sigma-1 inhibition reduced breast tumor cell viability in vitro and tumor growth in vivo [157].
**Transferrin Receptor (TfR)** 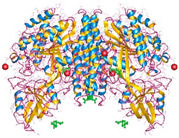	Iron uptake receptor highly expressed in proliferating cells.	Tf-conjugated liposomes and nanoparticles for chemotherapeutic delivery.	Enhances drug accumulation in tumor cells and reduces systemic toxicity.	Upregulated in breast tumors; associated with aggressive phenotype.	TfR-targeted liposomal doxorubicin improved therapeutic index in preclinical breast cancer models [79].
**Urokinase Plasminogen Activator Receptor (uPAR)** 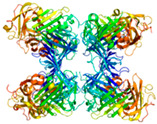	Regulates extracellular matrix degradation and cell migration.	Peptide-based ligands and antibody-drug conjugates targeting uPAR.	Suppresses invasion and metastasis.	Highly expressed in invasive breast carcinomas.	uPAR-targeted nanoparticles reduced metastatic burden in breast cancer xenografts [158].
**Transmembrane**					
**Biotin Receptor** 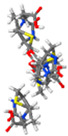	Overexpressed in many cancers.	Biotin-targeted liposomes improve drug delivery specificity.	Enhances specificity in drug delivery; minimizes off-target effects.	Increased expression in breast cancer enhances targeting capabilities.	Biotinylated liposomes have shown improved accumulation in breast cancer models [41].
**Cluster of Differentiation 44 (CD44)** 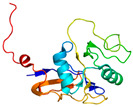	Involved in cell adhesion, migration, and proliferation.	CD44-targeted liposomes can reduce tumor growth and metastasis.	Inhibits tumor cell adhesion and migration; improves treatment efficacy.	CD44 is often overexpressed in breast cancer and is linked to aggressive behavior.	CD44-targeted therapies reduce tumor growth and improve survival rates in breast cancer models [159].
**Human Epidermal Growth Factor Receptors 2 (HER2)** 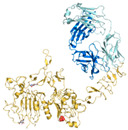	Receptor tyrosine kinase driving cell proliferation and survival.	Monoclonal antibodies (trastuzumab), antibody–drug conjugates, and HER2-targeted nanoparticles.	Improves survival and reduces recurrence in HER2+ breast cancer.	Overexpressed in ~20% of breast cancers; marker of aggressive disease.	HER2-targeted therapies have transformed prognosis in HER2+ breast cancer patients [160].
**Integrin Receptors** 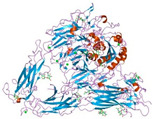	Mediators of cell adhesion, migration, and angiogenesis.	RGD peptide-modified nanoparticles and integrin-blocking antibodies.	Reduces angiogenesis and metastatic spread.	Integrin αvβ3 and α6β4 overexpressed in aggressive breast cancer subtypes.	Integrin-targeted drug delivery enhanced anti-tumor efficacy in breast models [161].
**Luteinizing Hormone-releasing Hormone Receptor (LHRHR)** 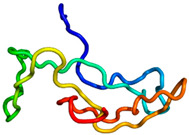	Involved in hormone regulation; overexpressed in some cancers.	LHRHR-targeted liposomes deliver drugs to hormone-responsive tumors.	Enhances targeting for hormone-dependent therapies; improves outcomes.	LHRHR is expressed in some breast cancer types, enabling specific targeting.	LHRH analogs have been shown to inhibit breast cancer cell growth in preclinical studies [162].
**Mucin 1 (MUC1)** 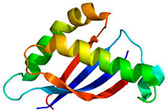	Transmembrane glycoprotein involved in cell adhesion and signaling.	MUC1-specific antibodies, vaccines, and nanocarriers.	Enables selective targeting and immunomodulation.	Overexpressed in >90% of breast carcinomas.	MUC1-targeted liposomes improved drug delivery in breast cancer xenografts [163].
**Neuropilin 1 (NRP1)** 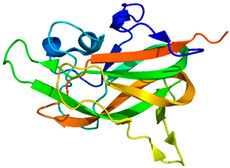	Involved in angiogenesis and tumor growth.	NRP1-targeted liposomes can inhibit blood vessel formation in tumors.	Reduces tumor growth and metastasis; disrupts angiogenic processes.	NRP1 is involved in the aggressive growth of breast tumors through angiogenesis.	NRP1 blockade has been shown to reduce tumor angiogenesis and growth in breast cancer studies [164].
**Internal Cell Receptor**					
**Estrogen Receptors (ERs)** 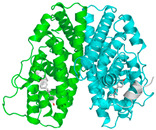	Mediate the effects of estrogen; involved in hormone-responsive breast cancer.	ER-targeted liposomes to deliver drugs specifically to estrogen-responsive tumors.	Enhances specificity of drug delivery; improves efficacy in ER-positive tumors.	ER is overexpressed in many breast cancers, making it a critical target for therapy.	ER-targeted therapies form backbone of hormone therapy in breast cancer [165].
**Progesterone Receptors (PRs)** 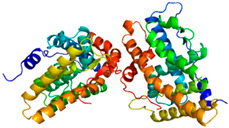	Mediate the effects of progesterone; involved in hormone-responsive breast cancer.	PR-targeted liposomes can improve drug delivery to progesterone-responsive tumors.	Increases drug concentration in PR-positive tumors; minimizes side effects.	PR expression is crucial in a subset of breast cancers, especially in ER-positive cases.	PR-targeted therapies enhance therapeutic efficacy in PR-positive breast cancer models [166].
**Enzyme**					
**Matrix Metalloproteinases (MMPs)** 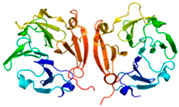	Proteolytic enzymes promoting extracellular matrix degradation and metastasis.	MMP inhibitors and MMP-cleavable prodrugs.	Limits invasion and metastatic dissemination.	MMP-2 and MMP-9 overexpressed in invasive breast cancers.	MMP-activated drug conjugates reduced metastasis in preclinical models [167]
**Secretory Phospholipase A2 (sPLA2)** 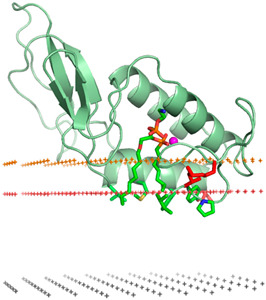	Involved in inflammation and tumor progression; associated with tumor microenvironment changes.	sPLA2-targeted liposomes can enhance drug delivery to tumors with high sPLA2 activity.	Increases drug accumulation in tumor sites; reduces side effects in healthy tissues.	High sPLA2 activity correlates with aggressive breast cancer phenotypes.	Targeting sPLA2 in liposomes has shown improved drug delivery and anti-tumor effects in breast cancer studies [168].

**Table 5:** Classifications of plant-based phospholipids.

Types	IUPAC Name	Sources	Synthesis	Application	References
**Phosphatidylcholine (PC)** 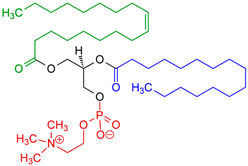	1,2-diacyl-sn-glycero-3-phosphocholine	Soybeans, sunflower seeds, mustard seeds, rapeseed, and palm oil.	Extraction from lecithin; lyso-PC generated enzymatically from PC when needed for derivatization (e.g., ethanolysis → 2-acyl-1-lyso-PC).	Commonly used in functional foods and pharmaceutical products, PC improves bioavailability and nutrient absorption. Palm oil-derived PC is especially valued for stability in emulsions and liposome formulations.	[171,172,173]
**Phosphatidylethanolamine (PE)** 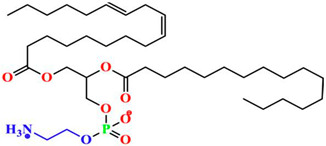	1,2-diacyl-sn-glycero-3-phosphoethanolamine	Soybeans, sunflower seeds, mustard seeds, and palm oil.	Biological routes (cellular): CDP-ethanolamine (Kennedy) pathway and mitochondrial phosphatidylserine decarboxylase (PISD/PSD) are the two major PE sources; lyso-PE acylation and head-group exchange are minor.	PE is essential in cell membranes for flexibility and permeability. Palm oil-derived PE is particularly useful in creating stable liposomes for drug delivery systems due to its membrane-assisting properties.	[174,175]
**Phosphatidylinositol (PI)** 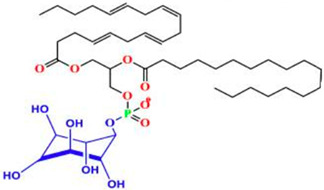	Phosphatidylinositol 4,5-bisphosphate	Soybeans, peanuts, legumes, and palm oil.	De novo PI synthesis at the ER: PA → CDP-DG → PI (via CDS1/2 and PI synthase), followed by acyl-chain remodeling; PLD transphosphatidylation can access PI from PC.	Involved in cellular signaling and beneficial in cosmetics due to its hydrating properties. PI from palm oil is also valued in food and pharmaceutical formulations where additional stability is desired.	[176,177,178]
**Phosphatidylserine (PS)** 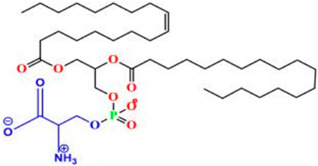	1,2-diacyl-sn-glycero-3-phospho-L-serine	Soybeans, sunflower seeds, and palm oil.	Dominant industrial method: PLD-mediated transphosphatidylation of PC with L-serine (aqueous/green solvent systems); avoids animal sources.	Known for supporting cognitive health, PS is popular in neuroprotective supplements. Palm oil-derived PS is used for its stability and bioavailability in cognitive support formulations.	[179,180]
**Phosphatidylglycerol (PG)** 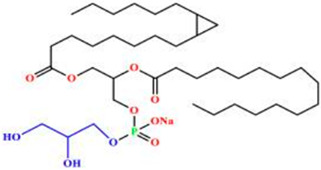	1,2-diacyl-sn-glycero-3-phospho-sn-glycerol	Soybeans, sunflower seeds, canola, chai seeds, flaxseeds, palm oil (not predominant).	Accessible via PLD-based transphosphatidylation from PC (rare phospholipids synthesis toolkit).	As emulsifier in dressings, sauces, and baked goods, helping to improve texture and stability. Also used as nutritional supplements for heart health and cholesterol management. In pharmaceuticals, it is used in liposomes to enhance the delivery of poorly soluble drugs. In cosmetics, PG provides hydration in moisturizers and stabilizes creams and lotions. It helps study lipid behavior and cell dynamics.	[169,181]
**Phosphatidic Acid (PA)** 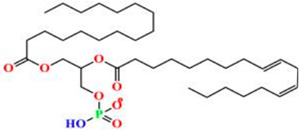	1,2-diacyl-sn-glycero-3-phosphate	Soybeans, mustard seeds, oil-rich seeds, and palm oil.	Formed by PLD hydrolysis of PC/other phospholipids; also via glycerol-3-phosphate acylation → LPA → PA (ER pathway).	A precursor to other phospholipids and key for cellular signaling, PA from palm oil is used in cosmetics and supplements for muscle support and cellular energy enhancement.	[182,183,184]
**Lysophosphatidylcholine (LPC)** 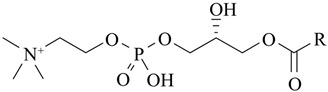	2-acyl-sn-glycero-3-phosphocholine (lyso-PC)	Sunflower seeds, rapeseed, soybeans, and palm oil.	Generated by hydrolysis/transesterification of PC (e.g., enzymatic ethanolysis to 2-acyl-1-lyso-PC) for further conjugation.	With strong emulsifying properties, LPC stabilizes emulsions in food products, cosmetics, and pharmaceuticals. Palm oil-derived LPC is particularly beneficial for applications requiring stable and long-lasting emulsions.	[185,186]
**Cardiolipin** 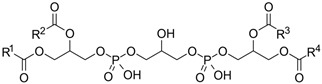	1,3-bis(sn-3′-phosphatidyl)-sn-glycerol	Wheat germ, soy lecithin, and palm oil (less commonly used).	Synthesized from PA within mitochondria; unique mitochondrial signature lipid (not typically plant-extracted for products). Its absence causes mitochondrial defects.	Important for mitochondrial health, cardiolipin is studied for energy metabolism and cellular health. Palm oil-derived cardiolipin is used in supplements focusing on mitochondrial function.	[187,188]

**Table 6:** Physical properties of phospholipids.

Types	Physical Properties				References
	Appearance	Texture	Solubility	Melting Point	
**Phosphatidylcholine (PC)**	Appears as a white or off-white powder or waxy solid. In its natural form within cell membranes, it appears as a component of lipid bilayers, contributing to membrane fluidity and structure.	Soft, waxy texture at room temperature. This texture aids in its ability to form smooth layers, which is critical for creating emulsions and liposomal structures.	Amphiphilic, meaning it has both hydrophilic and hydrophobic regions. It is soluble in organic solvents such as ethanol, chloroform, and methanol but is generally insoluble in water unless dispersed as part of an emulsion or liposome.	Tm depends on acyl chains: DSPC ~55 °C, DPPC ~42 °C, DMPC ~22 °C, egg PC < 0 °C	[189,190,191,192,193]
**Phosphatidylethanolamine (PE)**	Typically appears as a white or off-white powder when isolated; in natural forms, it may vary from colorless to pale yellow.	Powder or crystalline texture in purified form; waxy in a lipid mixture.	Poorly soluble in water but soluble in organic solvents like ethanol, chloroform, and methanol.	Generally, between 60 and 80 °C, though it can vary based on the fatty acid chains attached.	[175,194,195,196]
**Phosphatidylinositol (PI)**	Appears as a white or off-white powder when purified. In its natural state within lipid mixtures, PI can have a yellowish to pale brown hue, depending on its extraction source and the presence of any minor impurities.	A powdery texture in its isolated form but can also appear waxy or sticky in more concentrated lipid mixtures. This texture can influence its ease of handling and formulation, particularly when incorporating it into liposomal or emulsion systems for research and pharmaceutical applications.	Poorly soluble in water due to its amphipathic structure, which means it has both hydrophobic and hydrophilic parts. However, it is readily soluble in organic solvents like chloroform, ethanol, and methanol. This solubility characteristic makes PI ideal for extraction and purification through organic solvents and suitable for formulating lipid-based systems in non-aqueous environments.	Typically ranging from 55 to 75 °C, largely depending on the fatty acid composition. For example, PI molecules with more saturated fatty acid tails will have higher melting points, making them more stable at room temperature, whereas PI with unsaturated chains will have lower melting points and may be liquid at lower temperatures. This variability impacts its stability in formulations requiring specific thermal properties.	[197,198,199]
**Phosphatidylserine (PS)**	Appears as white to off-white, waxy solid or powder.	In its powder form, PS typically has a fine, dry texture. It may feel slightly greasy due to its lipid nature.	Sparingly soluble in water but is more soluble in organic solvents such as ethanol, chloroform, and methanol. It forms micelles or liposomes in aqueous solutions, which helps in its incorporation in biological systems.	The melting point can vary depending on its source and purity but typically ranges from 180 °C to 220 °C. The specific structure, such as the types of fatty acid chains attached, influences its exact melting point.	[179,194,200]
**Phosphatidylglycerol (PG)**	Appears as white to off-white or a waxy solid when it is isolated.	Variations in terms of a powdered texture to a slightly waxy form, based on the purity and form.	Sparingly soluble in water but it is soluble in other solvents such as the organic solvents namely the chloroform and ethanol which are used as a medium to dissolve lipids.	The melting point range is between 60 °C and 80 °C, which depends on the presence of the fatty acid chains.	[201,202,203,204,205]
**Phosphatidic Acid (PA)**	Appears as a white to slightly off-white powder or as a crystalline solid.	In the form of a purified powder or waxy.	Sparingly soluble in water but it is soluble in other medium of organic solvents such as methanol, ethanol, and chloroform.	The melting point range is between 55 °C and 65 °C, which varies with the presence of distinctive fatty acid chain lengths.	[182,206,207,208]
**Lysophosphatidylcholine (LPC)**	Appears as white or light yellowish in the form of a powder or waxy substance.	In the form of a purified powder or lightly waxy.	Amphiphilic, meaning it has a better solubility in water in contrast to other phospholipids as it has single fatty acid tail, and it has solubility properties in ethanol and chloroform.	The melting point range is between 20 °C and 40 °C, which varies with the presence of distinctive fatty acid components.	[209,210,211,212]
**Cardiolipin**	Appears as white to off-white in the form of a powder or waxy solid.	In the form of waxy or crystalline, based on its isolation and purity characteristics.	Insolubility in water but soluble in other solvent mediums such as ethanol, chloroform, and methanol.	The melting point range is between 60 °C and 70 °C, which varies with the presence of fatty acid components.	[213,214,215,216]

**Table 7:** Chemical properties of phospholipids.

Types	Chemical Properties							References
	Structure	Hydrophilic and Hydrophobic Regions	Acidic and Basic Properties	Oxidative Stability	Reaction to Heat	Interaction with Compounds	Hydration and Dehydration	
**Phosphatidylcholine (PC)**	Choline head group linked to phosphate and glycerol backbone with two fatty acyl chains	Hydrophilic choline–phosphate head; hydrophobic acyl tails	The phosphate group in PC is slightly acidic, but the choline moiety gives it an overall neutral or zwitterionic charge. This charge neutrality allows PC to interact with a wide range of molecules without strong electrostatic repulsion or attraction.	Stability against oxidation depends on the degree of unsaturation in its fatty acid chains. Unsaturated PCs are more susceptible to oxidation, potentially leading to rancidity.	Heat sensitive and can degrade at high temperatures, especially if it contains unsaturated fatty acids. Thermal degradation can affect its emulsifying and structural properties.	Interacts with proteins, carbohydrates, and other lipids, enhancing the stability and texture of emulsified products. It also interacts well with therapeutic compounds in liposomal formulations.	Can absorb water and form hydrated bilayers or vesicles. The bilayers can dehydrate and rehydrate without losing structural integrity, which is advantageous in liposome technology.	[189,190,191,192,193]
**Phosphatidylethanolamine (PE)**	Ethanolamine head group linked to phosphate and glycerol backbone with two fatty acyl chains	Polar ethanolamine–phosphate head; hydrophobic acyl tails	Typically acts as a weak base due to the amine group in ethanolamine, which can be protonated, increasing its positive charge under acidic conditions.	Moderate oxidative stability: susceptible to oxidation if it contains unsaturated fatty acid chains, though less stable than phosphatidylcholine due to fewer methyl groups.	Moderate stability to heat; can degrade or oxidize at higher temperatures, particularly in the presence of unsaturated fatty acids.	Easily interacts with other phospholipids and membrane proteins due to its amphipathic nature; stabilizes lipid bilayers in cell membranes.	It can undergo hydration and dehydration cycles; it can absorb water due to the polar head group, which is key in liposome and bilayer formation.	[175,194,195,196]
**Phosphatidylinositol (PI)**	Inositol head group linked to phosphate and glycerol backbone with two fatty acyl chains	Polar inositol–phosphate head; hydrophobic acyl tails	Generally neutral but exhibits weak acidity due to the phosphate group linked to the inositol ring. This phosphate group can donate hydrogen ions in slightly basic conditions, giving PI slight acidic properties that affect its charge and reactivity in the cellular environment. Additionally, the hydroxyl groups on the inositol ring provide additional polarity, enhancing its ability to form hydrogen bonds with surrounding molecules, especially within aqueous environments.	Moderate oxidative stability, with susceptibility to degradation if unsaturated fatty acids are present in its structure. The degree of unsaturation in the fatty acid chains significantly impacts its stability, as unsaturated chains are more prone to oxidation. This property is particularly relevant for PI’s application in liposome-based drug delivery, where oxidative stability can influence shelf-life and efficacy.	Stable under moderate heat but may begin to degrade at high temperatures, especially if the fatty acid tails contain unsaturated bonds. This thermal sensitivity makes PI suitable for some thermal processing methods, but caution is needed when exposed to prolonged or high heat to avoid lipid degradation, which could compromise its structural integrity in formulations.	Highly interactive with other molecules in cellular membranes and is essential in signaling due to its role as a precursor for phosphorylated derivatives, such as phosphatidylinositol phosphates (PIPs). These derivatives play significant roles in cell signaling pathways, particularly in response to external stimuli, and regulate various cellular functions like growth, metabolism, and survival.	It can attract water molecules, especially the PI’s inositol head group, which stabilizes within the cell membranes and undergoes interaction with signaling proteins. The impact of dehydration reduces the PI’s potential to interact with alternate molecules, hence impacting the stability of the membrane and leading towards the disruption of cell signaling pathways.	[197,198,199]
**Phosphatidylserine (PS)**	Serine head group linked to phosphate and glycerol backbone with two fatty acyl chains	Hydrophilic serine–phosphate head; hydrophobic acyl tails	Acidic by nature with a negative net charge at the physiological pH due to the presence of phosphate and carboxylate groups.	Susceptible to oxidation if unsaturated, hence affects the membrane integrity.	Stability is maintained at moderate temperature, but degradation occurs at high temperatures, which results in the breaking up the fatty acid chains and impacting its structure.	The interaction is strongly with ions specifically calcium, which aids the cell membranes to stabilize. Interaction with membrane proteins which plays a part in cellular signaling.	It can undergo hydration which is crucial for the maintenance of its position in the cell membrane. The alterations in the membrane fluidity and stability is because of dehydration.	[179,194,200]
**Phosphatidylglycerol (PG)**	Glycerol–phosphate head group linked to glycerol backbone with two fatty acyl chains	Polar glycerol–phosphate head; hydrophobic acyl tails	Slightly acidic due to the presence of the phosphate group with a negative charge at physiological pH.	Prone to oxidative degradation, specifically at the unsaturated fatty acid chains regions, which has a direct impact on the stability of the membrane.	Heat sensitivity, which leads towards the degradation of the fatty acid components at high temperatures, impacting the lipid bilayer initiations.	Interactions with ions, namely calcium and magnesium to form lipid bilayers that are stable combined with other phospholipids, essential for lung surfactant.	It can absorb water and results in swelling which is crucial for membrane formation. The reduction in the membrane fluidity and disruption of cellular functions is due to dehydration.	[201,202,203,204,205]
**Phosphatidic Acid (PA)**	Phosphate head group directly linked to glycerol backbone with two fatty acyl chains	Polar phosphate head; hydrophobic acyl tails	Strongly acidic; anionic phospholipid by nature with a negative charge due to the presence of the phosphate group.	Susceptible to oxidation, particularly if unsaturated fatty acids. Oxidation has a direct impact on the cellular signaling processes.	Relative stability at moderate heat; however, this results in degradation at high temperatures, impacting the structural integrity and acts as a signaling molecule.	Interaction with positive charged ions and proteins, significant in signaling pathways implied in cell growth and development.	Hydration at a quick speed, which is essential for the membrane structure. Dehydration leads to alterations in cell membrane stability and signaling activity.	[182,206,207,208]
**Lysophosphatidylcholine (LPC)**	Similar to PC but with one acyl chain	Hydrophilic choline–phosphate head; hydrophobic acyl tails	Zwitterionic; presence of both acidic and basic regions. The phosphate is acidic, and the choline is basic, with a net neutral charge at physiological pH.	Sensitivity to oxidation, specifically at the site of the single fatty acid chain, resulting in membrane destabilization.	Less stability in high temperatures, resulting in the degradation of the fatty acid chain and the loss of functionality as an emulsifier.	Good interaction with proteins and other lipids and involved in repairing of the cell membrane and signal transduction processes.	Distinctly hydrophilic and can absorb water, resulting in the formation of micelles in the aqueous mediums. Dehydration leads to the reduction in solubility and biological activity.	[209,210,211,212]
**Cardiolipin** **(CL)**	Two phosphatidic acids linked by glycerol; four acyl chains	Polar ethanolamine–phosphate head; hydrophobic acyl tails	Acidic by nature with a net negative charge due to the presence of two phosphate groups essential for its interactions in mitochondrial membranes.	Prone to oxidation, particularly in the mitochondrial membrane, leading to loss of functionality and linked with cell apoptosis.	Relatively stable at moderate temperatures, but undergoes degradation at high temperatures, particularly in the fatty acid chains, which affects mitochondrial membrane stability.	Strongly interacted with proteins and other lipids in the mitochondrial membrane, which is vital for energy production and enzyme activity.	Reduced hydration ability; potentiality to absorb water, which is crucial for maintaining mitochondrial membrane structure. The impact of dehydration leads to the concession of the membrane stability and the functionality of the embedded proteins.	[213,214,215,216]

## Data Availability

Not applicable.
